# Supplementary material for: Sporulation Activated via σW Protects Bacillus from a Tse1 Peptidoglycan Hydrolase Type VI Secretion System Effector
Source: Microbiol Spectr. 2023 Mar 14;11(2):e05045-22. doi: 10.1128/spectrum.05045-22 (PMC10100999; doi:10.1128/spectrum.05045-22)
Supplement: Supplemental file 10 — Fig. S1 to S9 and legends of Data Sets S1 to S9. Download spectrum.05045-22-s0001.pdf, PDF file, 0.9 MB [file spectrum.05045-22-s0001.pdf]

Title: Sporulation activated via σ^W protects *Bacillus* from a Tse1 peptidoglycan hydrolase T6SS effector

Authors: Alicia I. Pérez-Lorente[1], Carlos Molina-Santiago[1], Antonio de Vicente[1] and Diego Romero[1, *]

Supplementary Material

Supplementary Figures

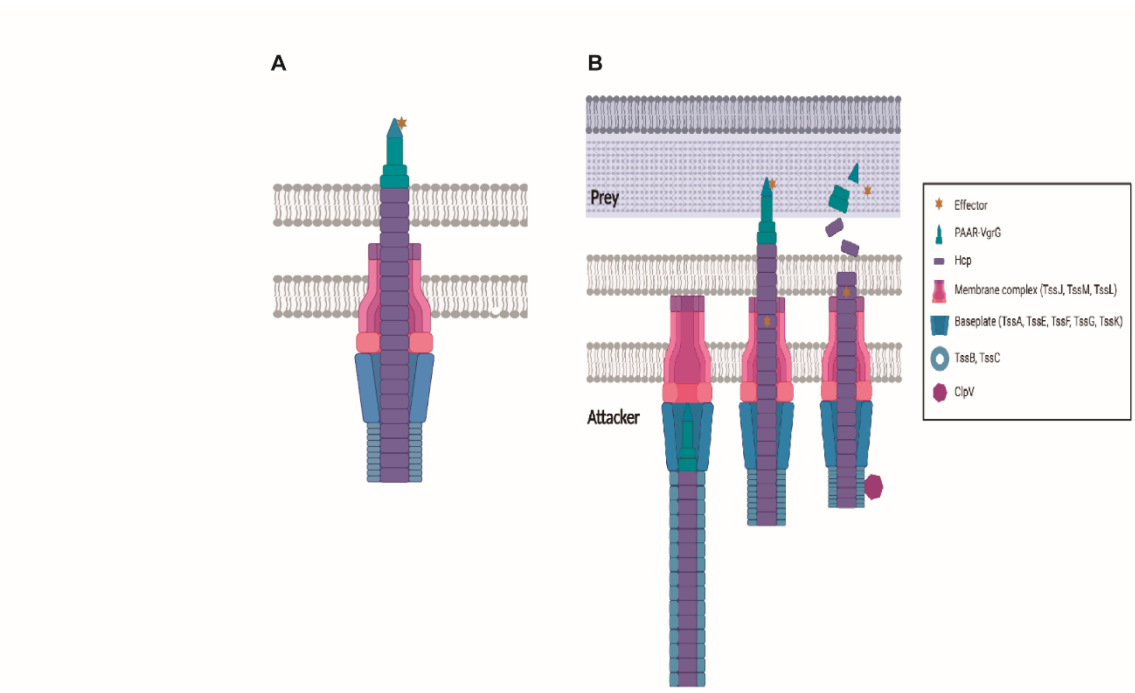

Figure S1. A) Schematic representation of T6SS structure. **B)** Representations of T6SS structure during extended, contraction, and disassembly states. The baseplate components (TssA, TssE, TssF, TssG, and TssK) are coloured in blue, the membrane complex (TssJ, TssM, and TssL) in pink and light purple, Hcp monomers in purple, the sheath components (TssB and TssC) in light blue, the spike (VgrG and PAAR) in green, and ClpV in strong purple.

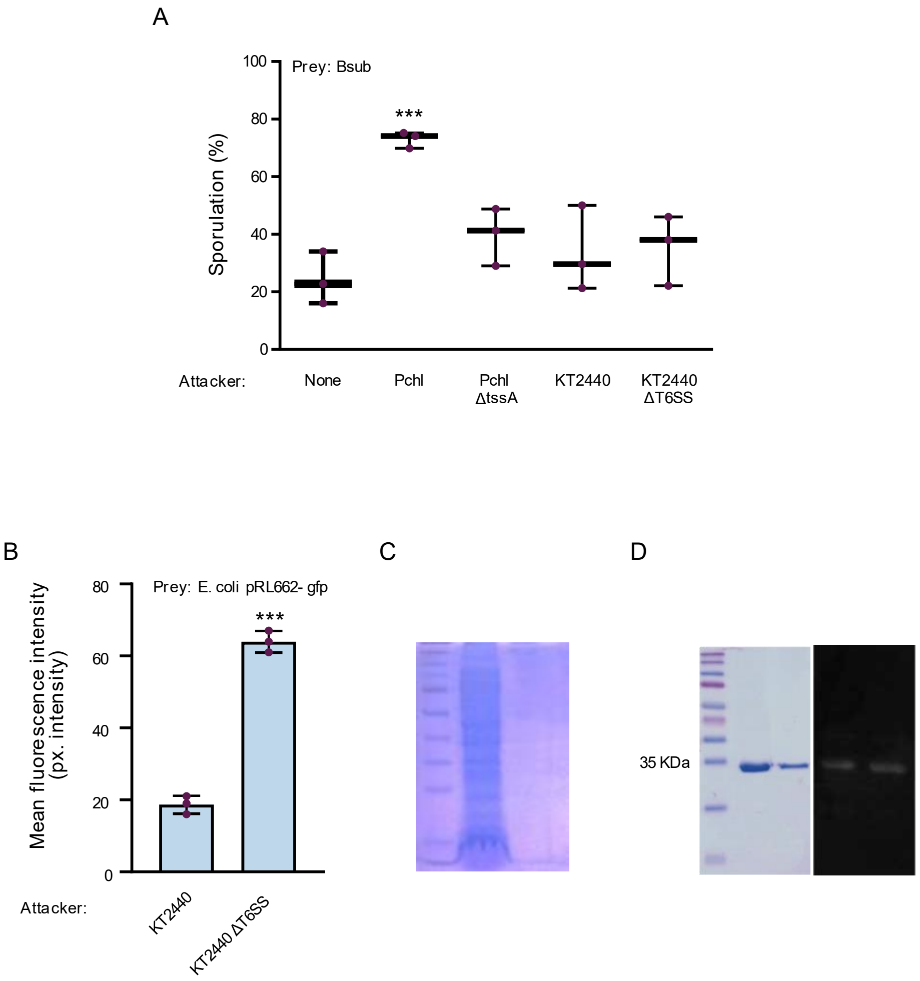

Figure S2. A) Percentages of Bsub sporulation in competition assays using different *Pseudomonas* strains as attackers (*P. chlororaphis* or *P. putida*) demonstrated that *P. putida* strains failed to induce Bsub sporulation **B)** Measurements of the fluorescence signal from *E. coli* pRL662-gfp after incubation with *P. putida* with (KT2440) or without (KT2440 ΔT6SS) a functional T6SS. KT2440 retained toxicity against *E. coli* while KT2440 ΔT6SS failed to kill *E. coli*. **C)** Gel electrophoresis of a clear lysate of *E. coli* containing the empty plasmid (line 2) and the elution fraction after passing the lysate through the HisTrap HP 5mL column (line 3). **D)** Gel electrophoresis of purified His-tagged Tse1 stained with Coomassie and a western blot of purified His-tagged Tse1 exposed to anti-His antibodies (1:1000). Experiments have been repeated at least three times with similar results. Gel images have been cropped and spliced for illustrative purposes. Statistical significance was assessed via t-tests. ***p value < 0.001.

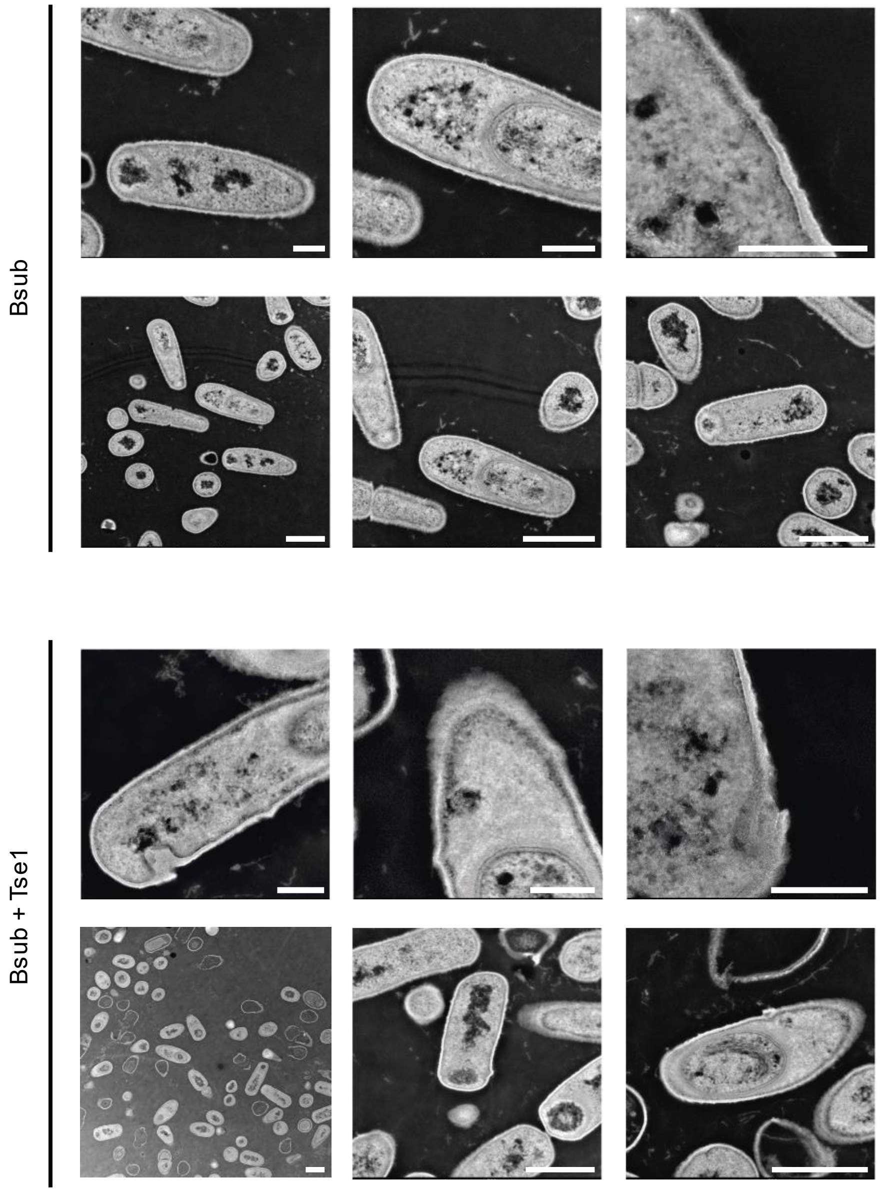

Figure S3. Transmission electron micrographs of Tse1-treated Bsub cells (bottom panels) showed cell wall protuberances and membrane invaginations compared with untreated cells (top panels). Scales bar of upper images of top and bottom panels equal 300 µm and scale bar of lower images of top and bottom panels equal 900 µm.

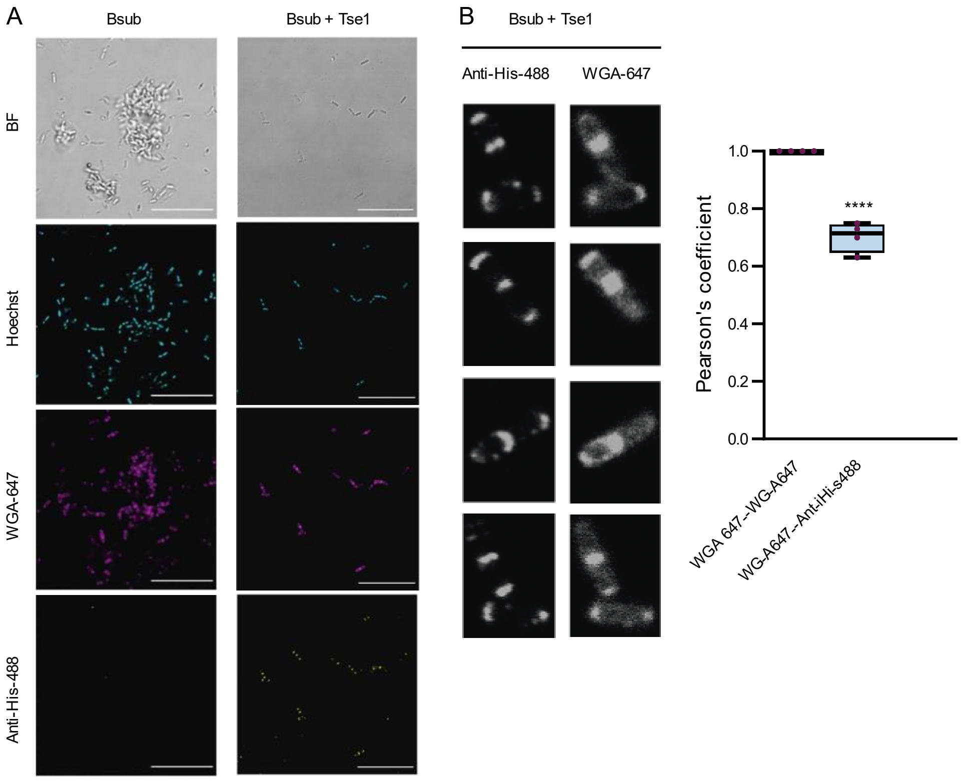

Figure S4. A) Immunofluorescence assays. CLSM of untreated (left panel) or Tse1-treated (right panels) *Bacillus* cells stained with Hoechst 33342 (Hoechst, blue channel) (DNA), WGA conjugated with Alexa Fluor 647 (WGA-647, pink channel) (peptidoglycan), or immunolabeling with anti-His antibody conjugated to Alexa Fluor 488 (Anti-His-488, yellow channel) (Tse1 detection). Scale bars equal 20 µm. **B)** Colocalization of Tse1 and peptidoglycan revealed via CLSM and immunodetection of Tse1 and WGA-647 staining. Pearson's correlation coefficient was calculated to evaluate colocalization between the two channels, Anti-His-488 and WGA-647. Colocalization analysis of the same channel (WGA-647) was used as a positive control for Pearson's correlation coefficient. N = 5 individual cells examined to calculate Pearson's correlation coefficient via FIJI/ImageJ software. Statistical significance was assessed via t-tests. ****p value < 0.0001.

PG

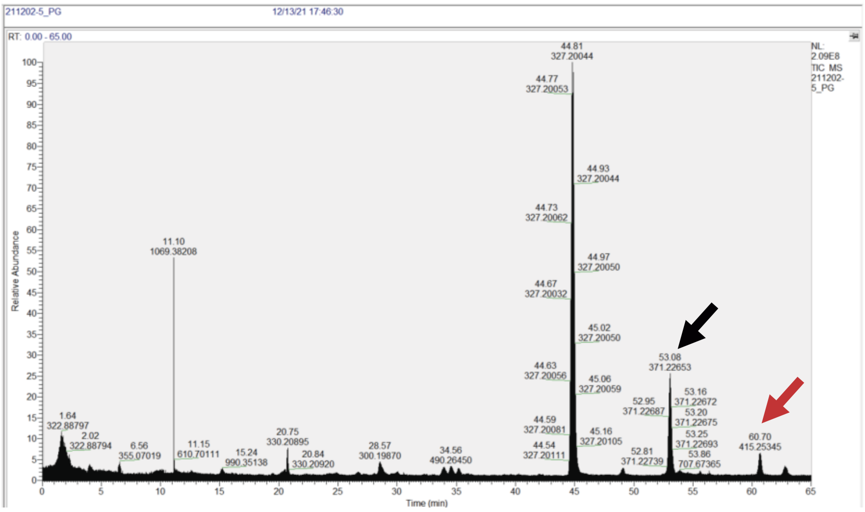

PG + Tse1

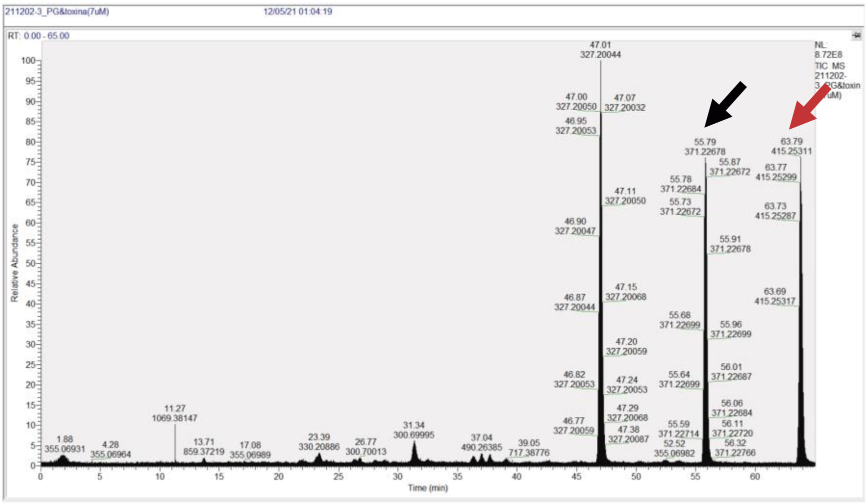

Figure S5. Relative abundance of the peaks found in mass spectrometry analysis of Bsub peptidoglycan treated with buffer (PG, top) or Tse1 (PG + Tse1, bottom). Black arrows designate the 371.23 m/z peak (NAG-NAM-Ala-Glu-mDAP-Ala) and red arrows designate the 415.25 m/z peak (disaccharide tetrapeptide NAG-NAM-Ala-Glu-mDAP). NAG, *N*-acetylglucosamine; NAM, *N*-acetylmuramic acid; Ala, alanine; Glu, glutamic acid; mDAP, *meso*-diaminopimelic acid.

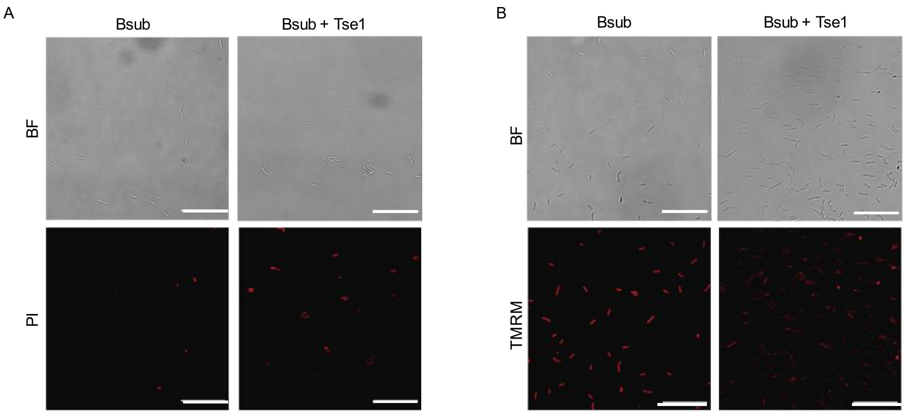

Figure S6. A) CLSM images of PI-stained Bsub cells showed a reduction of the mean fluorescence intensity, indicating an increase or permeabilized cells when Tse1 is added (right panels) compared to untreated cells (left panels). Broader field of **Fig. 4A**. **B)** CLSM images of TMRM-stained Bsub cells showing the membrane potential of untreated cells (top panels) or Tse1-treated Bsub cells (bottom panels). The data and images in **Fig. 4** are from the same experiment as the data displayed in this figure and were used to obtain the fluorescence intensity measures. Broader field of **Fig. 4B*.*** Scale bar equal 20 µm.

A

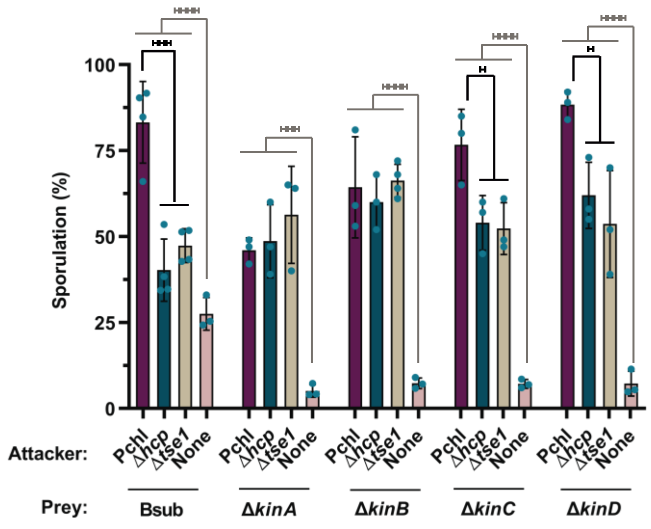

Figure S7. A) Competition assays between Bsub strains mutant for single kinases (*kinA*, *kinB*, *kinC,* or *kinD*) and Pchl strains (Pchl, Δ*hcp* and Δ*tse1*) showed that single *kinA* and *kinB* mutants are blind to the presence of a functional T6SS or Tse1. Statistical significance was assessed via t-tests. *p value < 0.1, ***p value < 0.001, ****p value < 0.0001.

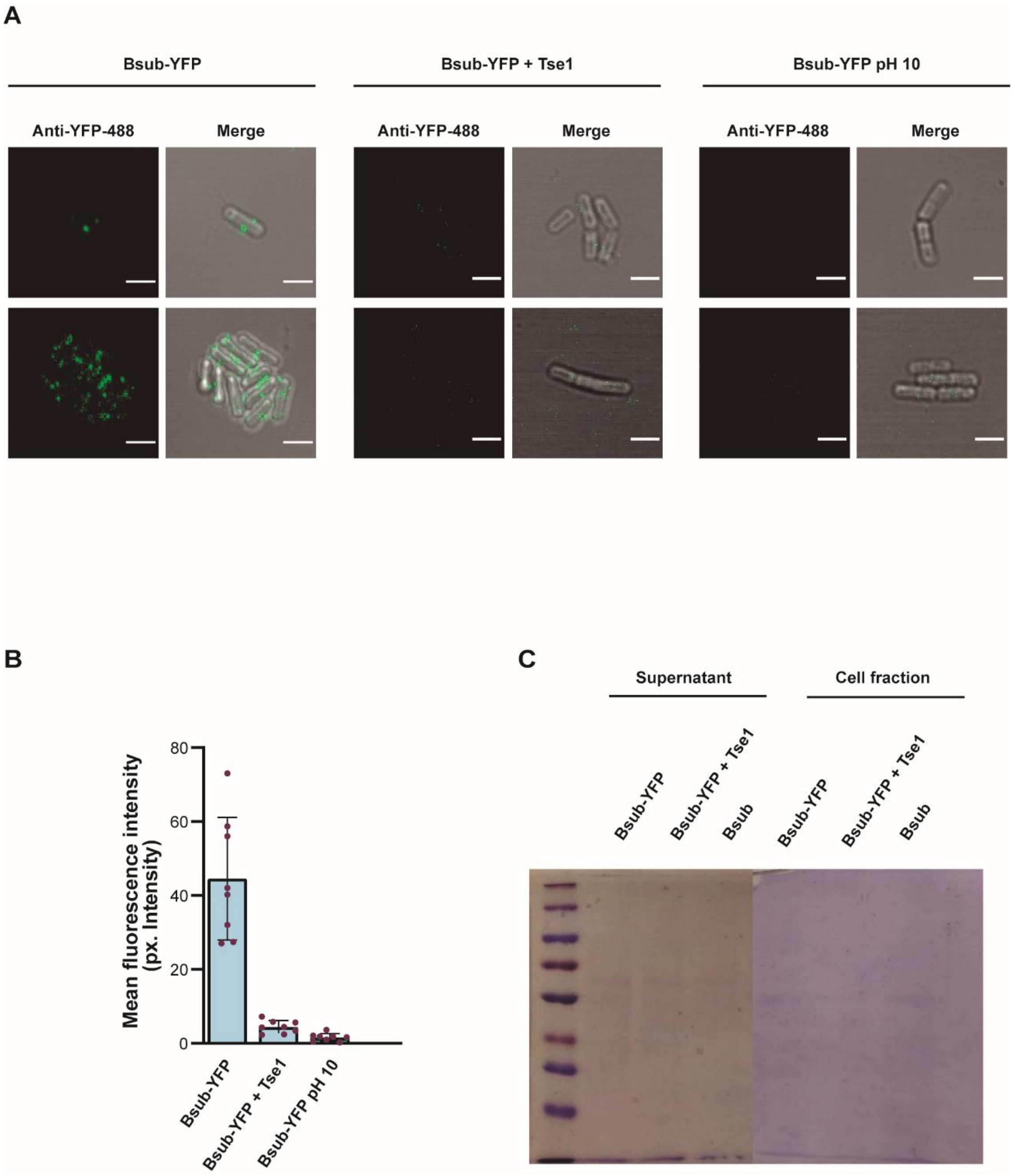

Figure S8. A) Additional images to **Fig 6D**. Immunofluorescence assay of *Bacillus* cells expressing RsiW-YFP translational fusion (Bsub-YFP) using anti-YFP antibody conjugated to Alexa Fluor 488 (green channel). Left panels: control Bsub-YFP cells. Central panels: Bsub-YFP cells treated with Tse1. Right panels: Bsub-YFP cells treated with buffer at pH 10 as positive control. Scale bars equal 2 µm. **B)** Quantification of the mean fluorescence intensity of analysed cells for each treatment shown of **Fig 6D**. N=8 for Bsub treated with Tse1, N=8 for untreated Bsub and N=8 for Bsub under alkaline

shock. N refers to the number of independent cells. **C)** Coomassie blue-stained SDS denaturing gel showing cell-free supernatant (left) or cell fractions (right) from WT *Bacillus* (Bsub) and *Bacillus* harbouring the RsiW-YFP translationalfusion either untreated (Bsub-YFP) or treated with Tse1 (Bsub-YFP + Tse1). Uncropped images of gel showed are shown in **Dataset S9.** Experiments have been repeated at least three times with similar results. Statistical significance was assessed via t-tests. ****p value < 0.0001.

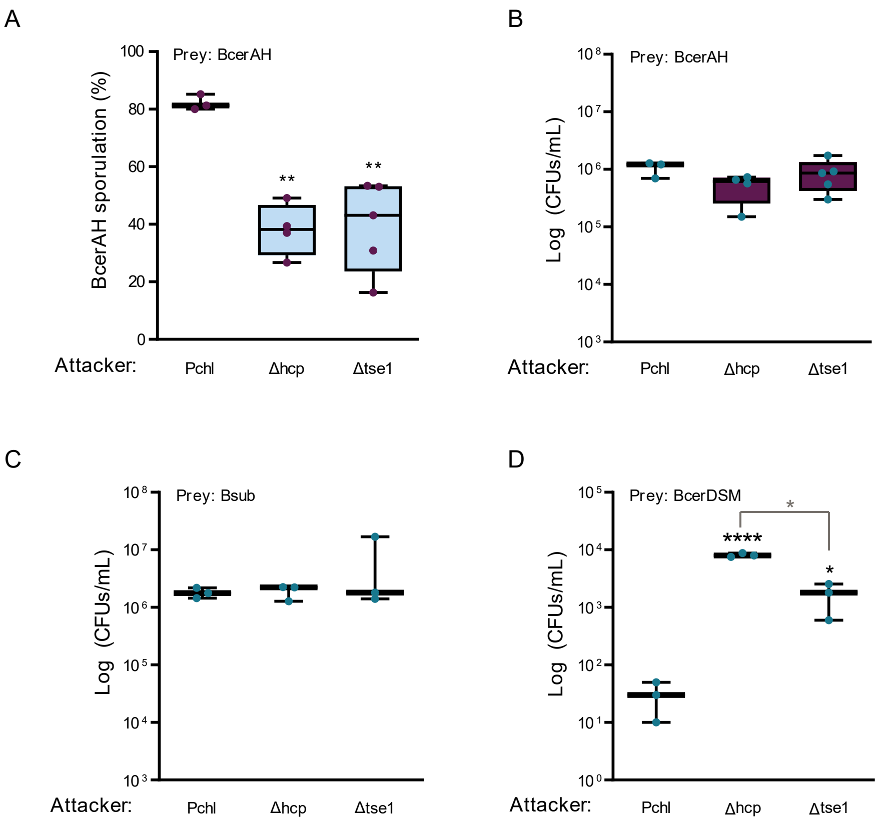

Figure S9. A) Percentages of *Bacillus cereus* (BcerAH) sporulation in competition assays with different Pchl strains as attackers (Pchl, Δ*hcp* and Δ*tse1*). **B-D)** Cell density (CFUs/mL) of different *Bacillus* strains (BcerAH, Bsub and BcerDSM) in competition assays with Pchl strains (Pchl, Δ*hcp* and Δ*tse1*). Statistical significance was assessed via t-tests. *p value < 0.1, **p value < 0.01, ****p value < 0.0001.

Supplementary Datasets

Dataset S1. Full names and accession numbers of strains used to generate T6SS phylogenetic tree.

Dataset S2. BLASTP analysis comparing Tse1 amino acid sequence against NCBI database.

Dataset S3. Upregulated genes after treatment with Tse1.

Dataset S4. Downregulated genes after treatment with Tse1.

Dataset S5. Raw luminescence data obtained for each sample and treatment.

Dataset S6. Strains used in this work.

Dataset S7. Oligonucleotides used in this study.

Dataset S8. List of raw CFUs (total and spore population) from all figures with sporulation percentage.

Dataset S9. Raw images of blots shown in Fig. 6E, Fig. S2D and Fig. S9C.
